# Ecologically relevant low oxygen levels reduce unfertilized oocyte output in *Caenorhabditis elegans*

**DOI:** 10.17912/micropub.biology.002266

**Published:** 2026-08-06

**Authors:** Emma R. Haxen, Mazharul Islam, Alexandra Grabowski, Sofie Mohlin, Nicole R. Posth, Sandeep Gopal, Emma U. Hammarlund

**Affiliations:** 1 Tissue Development and Evolution (TiDE), Department of Experimental Medical Science, Lund University, Lund, Sweden; 2 Lund Stem Cell Center, StemTherapy, Lund University, Lund, Sweden; 3 Department of Geosciences and Natural Resource Management, University of Copenhagen, Copenhagen, Denmark; 4 Lund University Cancer Center, Lund University, Lund, Sweden; 5 Lyman Briggs College and Department of Earth and Environmental Sciences, College of Natural Science, Michigan State University, East Lansing, Michigan, United States; 6 School of Earth, Environment, and Society and the Origins Institute, McMaster University, Hamilton, ON, Canada; 7 Division of Pediatrics, Department of Clinical Sciences, Lund University, Lund, Sweden

## Abstract

*Caenorhabditis elegans* naturally inhabits low-oxygen environments but is typically studied at 21% oxygen. We cultured adult *C. elegans* at 2.5%, 5%, and 21% oxygen during the fertile period, quantifying daily output of hatched larvae, unfertilized oocytes, and abnormal progeny (unhatched embryos, dead or deformed larvae). While we found no evidence for increased fertility at low oxygen levels compared to 21%, we observed a trend towards fewer unfertilized oocytes after peak fertility at 5% oxygen and significantly fewer at 2.5%. Our results indicate that reproductive output differs between laboratory conditions (high oxygen) and moderately low oxygen levels (closer to natural environments).

**Figure 1. Counts of hatched larvae and unfertilized oocytes at different oxygen levels:** Figure: Panels A, B, E, and F illustrate results from the experiment comparing reproductive output between 5% and 21% oxygen. Panels C, D, G, and H illustrate results from the experiment comparing reproductive output between 2.5% and 21% oxygen. Each data point represents one *C. elegans* hermaphrodite. For the experiment comparing 5% and 21% oxygen, total counts (A, E) quantify reproductive output during the first five days of adulthood. For the experiment comparing 2.5% and 21% oxygen, total counts (C, G) quantify reproductive output during the first six days of adulthood. For each experiment, data was pooled from three replicates (see Methods). **A** : Total counts of hatched, viable larvae per hermaphrodite at 5% and 21% oxygen. **B** : Daily production of hatched, viable larvae at 5% and 21% oxygen. **C** : Total counts of hatched, viable larvae at 2.5% and 21% oxygen. **D** : Daily counts of hatched, viable larvae at 2.5% and 21% oxygen. **E** : Total counts of unfertilized oocytes output per hermaphrodite at 5% and 21% oxygen. **F** : Daily output of unfertilized oocytes at 5% and 21% oxygen. **G** : Total counts of unfertilized oocytes at 2.5% and 21% oxygen. **H** : Daily output of unfertilized oocytes at 2.5% and 21% oxygen. Table 1: Summary statistics and test results for the comparison of reproductive output between oxygen levels. PS is the Mann–Whitney estimate (probability of superiority). Note, that the data represent pooling of multiple replicates (see Methods).

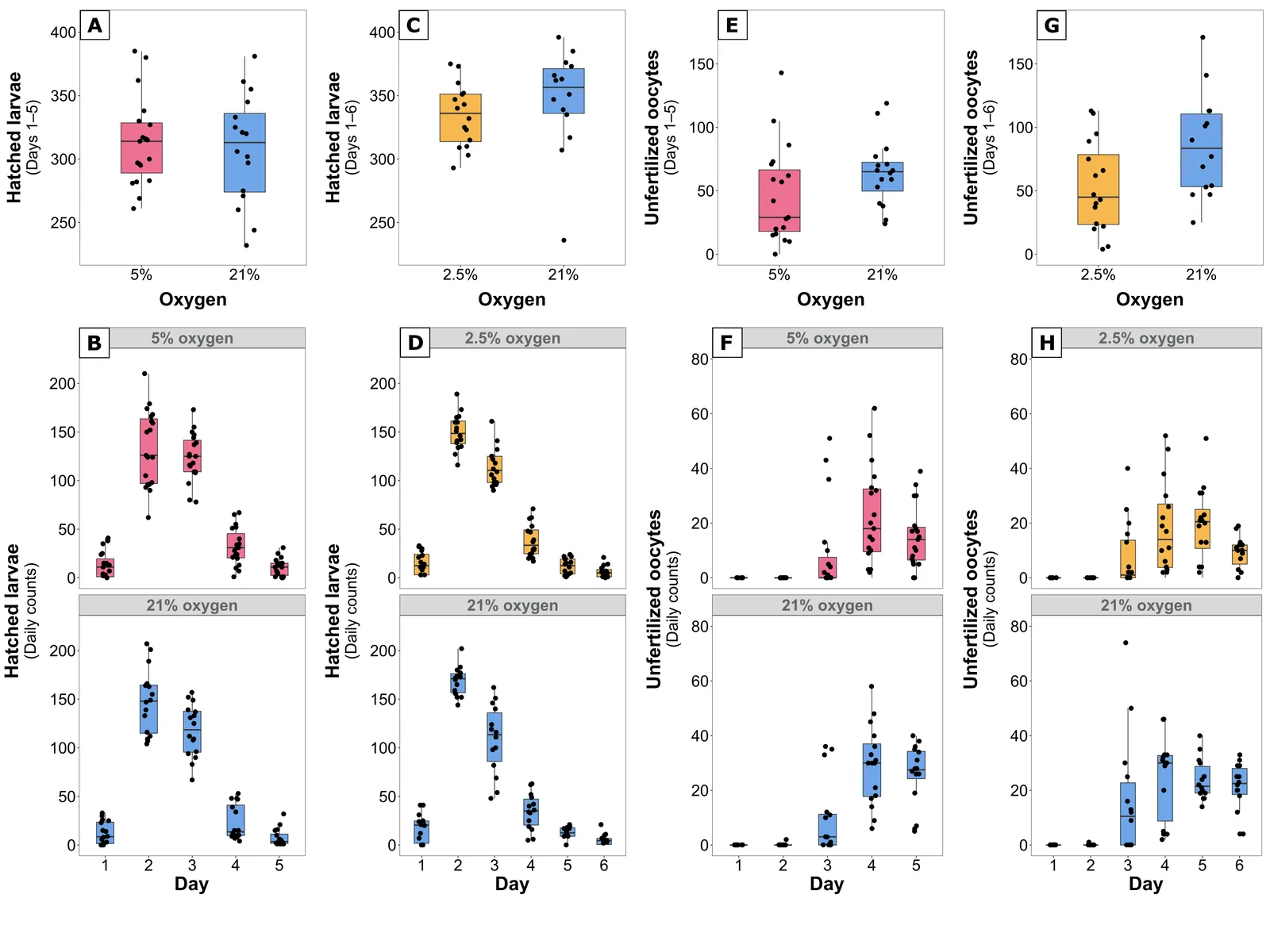



**Table:** 

Oxygen comparison	Period (days)	Measure	Test	Oxygen	n	Median (Range)	IQR	PS	98.33% CI	p-value
5% vs 21%	5	Hatched larvae	One-sided MWU	21%	16	313 (232–381)	62	0.508	0.305–1.000	0.475
5% vs 21%	5	Hatched larvae	One-sided MWU	5%	19	314 (261–385)	39.5			
5% vs 21%	5	Unfertilized oocytes	Two-sided MWU	21%	16	65 (24–119)	22.8	0.308	0.083–0.547	0.053
5% vs 21%	5	Unfertilized oocytes	Two-sided MWU	5%	19	29 (0–143)	48.5			
5% vs 21%	5	Abnormal progeny	Two-sided MWU	21%	16	3.5 (0–9)	4	0.569	0.329–0.782	0.503
5% vs 21%	5	Abnormal progeny	Two-sided MWU	5%	19	4 (0–12)	3.5			
2.5% vs 21%	6	Hatched larvae	One-sided MWU	21%	14	357 (236–396)	35.3	0.333	0.136–1.000	0.941
2.5% vs 21%	6	Hatched larvae	One-sided MWU	2.5%	16	336 (293–375)	37.5			
2.5% vs 21%	6	Unfertilized oocytes	One-sided MWU	21%	14	83.5 (25–171)	57.3	0.254	0.000–0.481	0.011
2.5% vs 21%	6	Unfertilized oocytes	One-sided MWU	2.5%	16	45 (4–113)	55			
2.5% vs 21%	6	Abnormal progeny	Two-sided MWU	21%	14	3.5 (0–15)	3.5	0.408	0.189–0.664	0.393
2.5% vs 21%	6	Abnormal progeny	Two-sided MWU	2.5%	16	2 (0–6)	2			

## Description

Critical developmental processes are associated with low tissue oxygen levels in many animal taxa (Simon and Keith, 2008; Mohyeldin et al., 2010; Carreau et al., 2011; Baccino-Calace et al., 2020; Brouillet et al., 2021; Carroll et al., 2021). However, less is known about tissue oxygen gradients and sensitivity in microscopic invertebrates that lack respiratory and circulatory systems. Many microscopic invertebrate species take up oxygen via simple diffusion across the body surface (Graham, 1988), meaning that their tissue oxygen gradients are directly coupled to ambient oxygen concentrations. Such animals are typically found in low-oxygen environments like sediment, soil, and compost (Jørgensen and Revsbech, 1985; Sexstone et al., 1985; Schratzberger et al., 2019; van den Hoogen et al., 2020), which are likely to result in low tissue oxygen levels. For example, the nematode *Caenorhabditis elegans* naturally inhabits rotting fruits and vegetation (Schulenburg and Félix, 2017), and this popular model organism has been shown to display a behavioral preference for 5–12% oxygen in laboratory studies (Gray et al., 2004). Yet, whether this preference for low oxygen levels is linked to oxygen sensitivity during reproduction and early development remains poorly understood.

The reproductive system of adult *C. elegans* hermaphrodites is known as “the germ line” and consists of two U-shaped gonadal arms connected by a common uterus (Hirsh et al., 1976). Each gonadal arm contains a pool of germline stem cells at its distal tip. As these germline stem cells proliferate, cells are pushed away from the stem cell niche in the proximal direction and begin to differentiate, eventually forming the oocytes that are passed through the spermatheca and fertilized (Hirsh et al., 1976; Albert Hubbard and Schedl, 2019). The spermatheca contains the adult hermaphrodites' supply of sperm cells (“self-sperm”) produced during the late larval stages (Hirsh et al., 1976). After peak fertility during days 1–3 of adulthood, the store of self-sperm is nearly depleted, and reduced sperm signaling leads to decreased rates of oocyte maturation and ovulation, although unfertilized oocytes are expelled at a reduced rate (Ward and Carrel, 1979; Riddle et al., 1997; McCarter et al., 1999; Scharf et al., 2021). The current study aims to explore the effects of moderately low oxygen levels on the reproductive output of *C. elegans* , including whether low oxygen levels lead to increased production of hatched, viable larvae throughout the entire fertile period (see Huang et al., 2004).

We cultured adult *C. elegans* hermaphrodites at 2.5%, 5%, and 21% oxygen and compared reproductive output between oxygen levels. Reproductive output was quantified as daily counts of (1) viable larvae; (2) unfertilized oocytes; and (3) abnormal progeny (unhatched embryos and dead or visibly deformed larvae, see Methods). We selected 5% oxygen because it falls within the range preferred by *C. elegans* (Gray et al., 2004) while approximating oxygen levels in mammalian stem cell niches (Mohyeldin et al., 2010, and references therein). We also quantified reproductive output at 2.5% oxygen, which is closer to tissue oxygen levels in many insect species (Birrell et al., 2024) and during mammalian embryonic development (Brouillet et al., 2021).

First, we compared reproductive output between adult hermaphrodites cultured at 5% and 21% oxygen over days 1–5 of adulthood to test whether more hatched, viable larvae would be produced at 5% oxygen. Median production of hatched, viable larvae was essentially identical between 5% and 21% oxygen, but statistical comparison was inconclusive regarding effects in the underlying population (probability of superiority, PS = 0.508; 98.33% CI = 0.305–1.000; one-sided exact p = 0.475; Fig. 1A ; Table 1). This reflected variability between replicates (PS per replicate: 0.680, 0.640, 0.389; see Methods). The observed daily distributions of larval counts appeared similar between oxygen levels, suggesting no major shift in the timing of peak fertility and generally similar daily fecundity between conditions ( Fig. 1B ). We found no significant difference in the production of abnormal progeny between 5% and 21% oxygen, but results were inconclusive regarding effects in the underlying population (PS = 0.569; 98.33% CI = 0.329–0.782; two-sided exact p = 0.503; Table 1).

To explore the effects of 5% oxygen on *C. elegans* germline function after peak fertility, we counted daily output of unfertilized oocytes over days 1–5 of adulthood. We observed a trend towards reduced total output of unfertilized oocytes at 5% oxygen (PS = 0.308; 98.33% CI = 0.083–0.547; two-sided exact p = 0.053; Fig. 1E, Table 1). This trend was consistent between replicates (PS per replicate: 0.160, 0.280, 0.352). No unfertilized oocytes were observed during the first two days of adulthood at either oxygen level, and output of unfertilized oocytes generally began on day three and increased on day four ( Fig. 1F ).

Next, we cultured *C. elegans* at 2.5% and 21% oxygen during days 1–6 of adulthood to test whether more hatched, viable larvae would be produced at 2.5% oxygen. We did not observe increased production of viable larvae at 2.5% oxygen (PS = 0.333; 98.33% CI = 0.136–1.000; one-sided exact p = 0.941; Table 1). Rather, median larval counts were reduced compared to 21% oxygen ( Fig. 1C ; PS per replicate: 0.278, 0.267, 0.460). As in the initial experiment, the daily distributions of larval counts appeared similar between 2.5% and 21% oxygen, suggesting no major shift in peak fertility timing ( Fig. 1D ). We observed no significant difference in the production of abnormal progeny between oxygen levels, but results were inconclusive regarding effects in the underlying population (PS = 0.408; 98.33% CI = 0.189–0.664; two-sided exact p = 0.393; Table 1).

Given the observed trend towards a reduced number of unfertilized oocytes at 5% oxygen, we investigated whether unfertilized oocyte output would be reduced at 2.5% compared to 21% oxygen. We observed a significant reduction in the total output of unfertilized oocytes when culturing *C. elegans* hermaphrodites at 2.5% oxygen during days 1–6 of adulthood (PS = 0.254; 98.33% CI = 0.000–0.481; one-sided exact p = 0.011; Table 1; Fig. 1G ). Again, output of unfertilized oocytes began on day three of adulthood and daily output after peak fertility was generally higher under 21% oxygen ( Fig. 1H ). Median output of unfertilized oocytes was lower at 2.5% oxygen in all replicates (PS per replicate: 0.375, 0.133, 0.080). Thus, our results indicate a reduction in unfertilized oocyte output at 2.5% compared to 21% oxygen.

To summarize, we did not find evidence to support increased fecundity of *C. elegans* hermaphrodites at 2.5% and 5% oxygen. Statistical comparisons of viable progeny production between oxygen levels were inconclusive but observed medians and effect size estimates suggested broadly similar fertility between 5% and 21% oxygen. Our findings complement the results of Freyth et al. (2010), who reported that *C. elegans* fertility is largely unchanged between ~10% and 21% oxygen, increased at 3.6% oxygen, and reduced at <3.6% oxygen. However, whereas Freyth et al. (2010) reported the total number of larvae counted after the first three days of adulthood, our daily counting protocol necessitated brief (10–15 min) daily exposures to 21% oxygen, which may have obscured any small fertility increase at 5% oxygen. Conversely, at 2.5% oxygen, we observed a trend towards reduced median production of hatched, viable larvae in all replicates, consistent with the results of Freyth et al. (2010). Decreased production of hatched larvae at 2.5% oxygen may reflect metabolic effects, as *C. elegans* has been shown to maintain stable respiration between ~4% oxygen and atmospheric levels (Anderson and Dusenbery, 1977; Van Voorhies and Ward, 2000).

In contrast to the inconclusive effects on fertility, lower oxygen levels consistently led to reduced output of unfertilized oocytes. Such a reduction would be expected if lowering ambient oxygen levels resulted in reduced rates of oocyte production. Oxygen-sensitive gamete differentiation rates would be consistent with the observation that mammalian cell differentiation is affected by oxygen levels (Ezashi et al., 2005; Berniakovich and Giorgio, 2013; Drela et al., 2014), and with the role of mitochondrial maturation and production of reactive oxygen species (ROS) in driving *C. elegans* oocyte differentiation (Charmpilas and Tavernarakis, 2020). However, reduced overall rates of oocyte production would lead to shifts in the timing of peak fertility under 2.5% and 5% oxygen compared to 21%, which we did not consistently observe (Figs. 1B,F). Thus, our results indicate that moderately low oxygen levels lead to reduced output of unfertilized oocytes after peak fertility, compared to standard laboratory conditions. Further studies are needed to determine the effects of oxygen on different germline functions in *C. elegans* .

## Methods

***C. elegans* maintenance**

The laboratory reference strain (Wild-type, Bristol) was cultured on nematode growth medium (NGM) in 60 mm vented petri dishes seeded with *Escherichia coli* ( OP50 strain) bacterial lawns. The NGM was prepared as detailed in Brenner (1974). Animals were maintained at 20°C for at least two weeks without starvation or overcrowding before being used for experiments.

**Controlling oxygen tensions**

During experiments, animals were cultured in a temperature-controlled laboratory under different oxygen pressures within custom-built hypoxia boxes. At the front, the boxes had a hinged door with magnet strips glued along the edges, connecting to magnet strips around the box opening. At the back, holes were drilled and fitted tightly with connectors to gas tubes and oxygen sensors. In the hypoxia boxes, oxygen levels were maintained using ProOx 110 Compact oxygen Controllers (BioSpherix, Ltd., RRID: SCR_021129), which kept oxygen concentrations at the set low levels by infusing the semi-sealed boxes with a gas mix consisting of 420 ppm CO _2_ in N _2_ . In the otherwise identical control chamber (21% oxygen), the sensor hole at the back of the box was left open to ambient air. Each box contained a fan to avoid oxygen gradients, as well as a beaker of Milli-Q water to ensure comparable humidity levels between the hypoxia and control boxes. Temperature and relative humidity levels were monitored using data loggers (HOBO® MX1101 Temp/RH Data Logger and HOBO® Pendant® MX Temp/Light (MX2202) Logger).

**Quantifying daily reproductive output**

Experiments quantifying reproductive output were initiated using animals at larval stage L4. Each experimental replicate was initiated by picking 10–20 L4 larvae onto an empty NGM plate. These L4 larvae were then distributed through blinded randomization between an equal number of NGM plates, half of which (for the low-oxygen group) had been left to degas oxygen in the hypoxia box overnight. After picking one L4 larva onto each NGM plate for the experiment, the plates were returned to the hypoxia and control boxes.

The initial experiment comparing reproductive output between 5% and 21% oxygen was conducted over days 1–5 of adulthood. The subsequent experiment to compare reproductive output between 2.5% and 21% oxygen was conducted over days 1–6 of adulthood. Daily transfers to fresh NGM plates necessitated brief (10–15 minutes) exposure to ambient conditions (21% oxygen), resulting in oxygen fluctuations similar to variability in the natural habitats of *C. elegans* . Every day during the study period, the animals were transferred to new NGM plates seeded with 100 µl OP50 lawns (the low-oxygen plates having been left to degas oxygen overnight). Blinded, randomized counting of hatched larvae, unfertilized oocytes, and abnormal progeny began on the third day of the experiments (counting reproductive output in the “Day one” plates) and ended on the seventh or eighth day (counting the “Day five” and “Day six” plates, respectively). Any egg that had not hatched after 48 hours was counted as unhatched. These were included in counts of abnormal progeny along with larvae with obvious morphological and locomotory defects (e.g., partial paralysis, large kinks or lumps). Unfertilized oocytes were identified based on their characteristic morphology, namely the lack of an eggshell, their brownish color, and their soft, disk-like shape (see Ward and Carrel, 1979; images in Kern et al., 2021). Reproductive output from hermaphrodites that died during the course of the experiment were excluded from the analyses, leading to different sample sizes between oxygen levels in some replicates (see Statistics).

**Statistics**

Effects of low oxygen levels on reproductive output were investigated via two separate experiments, one comparing reproductive output between 5% and 21% oxygen, the other comparing output between 2.5% and 21% oxygen. Three experimental replicates were conducted for each comparison. Reproductive output was compared between low-oxygen (2.5% or 5% oxygen) and standard laboratory conditions (21% oxygen; control level) using Mann Whitney U testing. A non-parametric test was chosen due to the small sample size and different distributions of discrete count data between oxygen levels. In the initial comparison between 5% and 21% oxygen, two replicates had n = 5 hermaphrodites for each oxygen level, whereas the third replicate had n = 6 in the control group (21% oxygen) and n = 9 in the low-oxygen group (5% oxygen). In the subsequent comparison between 2.5% and 21% oxygen, n ranged from 3–6 in the control group and from 5–6 in the low-oxygen group. The uneven number of animals between oxygen levels was due to some hermaphrodites dying during the course of the experiments. After pooling the replicate data, we computed exact p-values, effect size estimates (probability of superiority, PS), and associated confidence intervals using the wmwTest() function from the asht package (version 1.0.1) in R (version 4.5.0). A fixed random seed was set to ensure reproducibility (set.seed(123)). We applied Holm–Bonferroni correction to control family-wise error rate over three pairwise comparisons (hatched larvae, unfertilized oocytes, and abnormal progeny), reporting 98.33% CIs for effect sizes.

## Reagents

**Table:** 

**Strain**	**Genotype**	**Source**
N2	*Caenorhabditis elegans* , wild type, Bristol.	CGC
OP50	*Escherichia coli* OP50	CGC
